# A Survey of Dental Caries Segmentation and Detection Techniques

**DOI:** 10.1155/2022/8415705

**Published:** 2022-04-11

**Authors:** Vincent Majanga, Serestina Viriri

**Affiliations:** Statistics and Computer Science, University of KwaZulu-Natal, Durban 4000, South Africa

## Abstract

Dental caries detection, in the past, has been a challenging task given the amount of information got from various radiographic images. Several methods have been introduced to improve the quality of images for faster caries detection. Deep learning has become the methodology of choice when it comes to analysis of medical images. This survey gives an in-depth look into the use of deep learning for object detection, segmentation, and classification. It further looks into literature on segmentation and detection methods of dental images through deep learning. From the literature studied, we found out that methods were grouped according to the type of dental caries (proximal, enamel), type of X-ray images used (extraoral, intraoral), and segmentation method (threshold-based, cluster-based, boundary-based, and region-based). From the works reviewed, the main focus has been found to be on threshold-based segmentation methods. Most of the reviewed papers have preferred the use of intraoral X-ray images over extraoral X-ray images to perform segmentation on dental images of already isolated parts of the teeth. This paper presents an in-depth analysis of recent research in deep learning for dental caries segmentation and detection. It involves discussing the methods and algorithms used in segmenting and detecting dental caries. It also discusses various existing models used and how they compare with each other in terms of system performance and evaluation. We also discuss the limitations of these methods, as well as future perspectives on how to improve their performance.

## 1. Introduction

For easier segmentation and detection of dental caries, prior knowledge for several tasks is needed. There is a need to understand the various sections of the tooth and the specific position of the lesion on the tooth. An understanding of the types of dental images to be used, for instance, panoramic or bitewing radiographs, is also needed. Furthermore, the specific regions or areas of interest which are required should be clear in order to be able to choose the suitable method for segmentation and detection of caries. All this information is required in order to achieve high performance segmentation and detection of dental caries.

### 1.1. Tooth Anatomy

**Figure 1:**
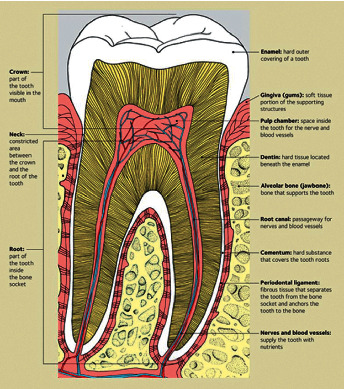
Cross section of a molar tooth [2].

The tooth is a small white structure found in the jawbone of animals and human beings. In human beings, the number of teeth ranges from 20 primary teeth in children to 28–32 permanent teeth in adults. Further, the tooth can be broken down into three main layers: the crown, neck, and root. The crown is the visible region above the gum line. The neck connects the crown to the root, while the root is the region inside the bone socket [1]. Additionally, each tooth consists of hard tissues that protect the soft tissues in the center, as can be seen in Figure 1.

### 1.2. Dental Caries

Dental caries is a tooth infection caused by bacteria. Diet that includes fructose, sucrose, and glucose accelerates the occurrence of dental caries. The acid released from the above process leads to demineralization of the tooth surface. Caries occurs when the rate of demineralization is less than the rate of decay. There are various categories of dental caries and these can be characterized [3]. The protocol characterizes dental caries based on its location and also the affected tooth [4]. The categories are as follows:

  Class I: dental caries on occlusal surfaces of posterior teeth, for instance, molars and incisors.
  Class II: occurs on proximal surfaces of posterior teeth.
  Class III: occurs on interproximal surfaces of anterior teeth, with no incisor edge involvement.
  Class IV: occurs on interproximal surfaces of anterior teeth with incisor edge involvement.
  Class V: occurs on the lingual or cervical third of the facial surface of the tooth.
  Class VI: occurs on the occlusal or incisor edge, worn away due to attrition.

From positional classification, caries can also be classified based on the severity of lesions on the tooth. This is done based on the amount of dentin and enamel that has been affected by the caries.

  Incipient caries are caries that have a depth of less than half of the enamel of the tooth.
  Moderate caries are caries that are more than halfway through the enamel but do not touch the dentin.
  Advanced caries are caries that extend to the dentin region.
  Severe caries are caries that extend more than halfway through the dentin and even reach the pulp.

Identification of caries under classes I, IV, and VI can be done during clinical inspection, since the regions are visible orally. The introduction of X-rays in the medical field has greatly improved diagnosis of various ailments. In the dental field, radiography has improved the visual inspection of patient's teeth. X-rays have enabled professionals to be able to view previously unobservable regions of caries that would have gone untreated.

### 1.3. Dental Radiographs: X-Rays

**Figure 2:**
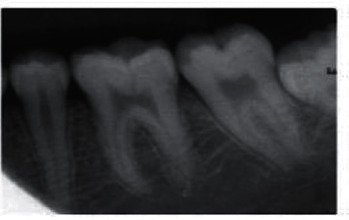
Periapical radiograph.

**Figure 3:**
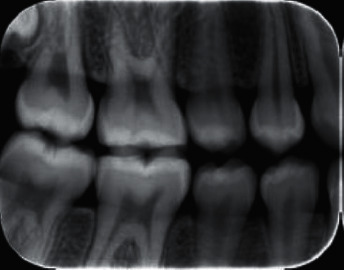
Bitewing radiograph.

**Figure 4:**
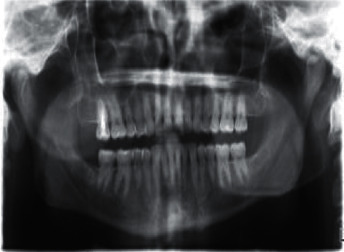
Panoramic radiograph.

There are varying degrees of information needed depending on the form of treatment required to diagnose a certain ailment. An X-ray or radiograph is a digital film that represents unobservable information not visible by the naked eye. Figures 2–4 show some types of radiographs. The work in [5] explains three types of X-rays that are commonly used to diagnose dental health. There are two types of radiographs, intraoral and extraoral [6].

  Intraoral radiographs: X-ray film captures radiographic images while inside the mouth. This type is subdivided further into *bitewing radiographs*, *periapical radiographs*, and *occlusal radiographs*.
  Extraoral radiographs: X-ray film captures radiographic images while outside the mouth. This type is subdivided further into *panoramic radiograph*, *computed tomography (CT)*, and *sialography*.

## 2. Deep Learning Systems Application Areas

The introduction of deep learning systems has contributed to several application areas using medical images.

### 2.1. Digital Microscopy and Pathology

Deep learning methods have been very popular in these areas, especially with the growing availability of tissue specimen. In this domain, deep learning techniques developed have had their main focus on three broad challenges. These are segmentation of large organs, detecting, segmenting, and classifying nuclei, and also detecting and classifying region of interest of lesions. Other areas where deep learning techniques have contributed include normalizing histopathology and color normalization in image analysis. The work in [7] introduces a method for normalizing stains on histopathology images using autoencoders. Color normalization is further demonstrated by [8], where the use of convolutional neural networkss (CNN) for tissue classification in stained images was introduced. *Brain*. In this area, deep neural networks (DNN) have been used for brain image analysis in several domains, most notably the classification of Alzheimer's disease. Other domains include segmentation of brain tissues and anatomical structures, for instance, hippocampus. There are also the segmentation and detection of brain tumor, lesions, and microbleeds. There are other tasks that require more anatomical information like white matter lesion segmentation, and [9] tackled such scenarios. They lowered the sampling rate of nonuniformed sampled patches to cover a larger part of the region of interest.

*Chest*. Deep learning also has contributed to thoracic image analysis, on both tomography and radiology. Deep neural networks have addressed the detection, characterization, and classifying nodules from radiology tests conducted. Computed tomography (CT) scans have detected several diseases, including lung diseases, from a single image. Chest radiography is the most common radiology test and therefore uses a large set of images that are used to train systems. These systems can be a combination of convolution neural networks used for image analysis and recurrent neural networks used for text analysis.

*Eye*. Deep learning algorithms have also been introduced in the analysis of eye images and have seen CNNs being employed to address the segmentation of anatomical structures. These networks have also addressed the detection and segmentation of retinal diseases, diagnosis, and assessment of image quality. The work in [10] shows the performance of a Google Inception v3 network for diabetic retinopathy detection and compares the results with those of seven ophthalmologists.

*Musculoskeletal*. In this domain, deep learning algorithms are used for identification and segmentation of joints, bones, and soft tissues in images. The method used in [11] is one of the applications that trained their system with musculoskeletal disc images and had very good performance across some radiology scoring tasks.

*Breast*. Research on breast imaging has resulted in [12], which shows significant advances over the state of the art, achieving performance of human readers on the region of interest (ROI). The main task also is to detect breast cancer, and this consists of several subtasks. These include detection and classification of lesions, micro classification, and cancer risk scores of images. The availability of huge amounts of image data has made mammography easier to perform and the most common method used for breast radiography.

*Abdomen*. Research on the abdomen is localized on the segmentation of organs, mainly pancreas, kidneys, liver (tumors), and bladder. The main radiograph used for most organs is the CT radiograph, as well as MRI only for prostate analysis. The method used by [13] introduces a hybrid neural network used to extract features that will be used further for classification. *Cardiac*. Cardiac image analysis has embraced deep learning in segmentation, tracking, classification, and accessing image quality. The MRI is used for radiographic testing. Reference [14] introduced the use of neural networks to segment the left ventricle using the recurrent connection of U-network architecture. Reference [15] used neural networks to perform regression for identifying some cardiac sequence on its model.

## 3. Factors That Lead to Dental Caries

[The biology, prevention, diagnosis and treatment of dental caries Scientific advances in the United States] show factors affecting caries, namely, saliva, bacteria, diet, and hereditary.

*Bacteria*. Reference [16] shows how plaques form around the surface of the tooth and then come into contact with carbohydrates to form acids that dissolve the tooth structure. This concept formed the basic foundation of dental caries. There is no specific way of how bacteria affects the tooth; thus it is hard to control the dental caries disease. In the method [17], how dental caries occurs from bacteria associating with fermented carbohydrates is explained, thus being referred to as a diet-bacteria disease. Researchers have associated the caries process with fermented carbohydrates [18] and showed a relationship between sugar and caries leading to acids formed on the tooth's surface. Rather, [19] demonstrated how frequent sweet snacks consumption is related to dental caries.

*Saliva*. It plays a critical part in the well-being of soft and hard tissues of teeth inside the mouth [20]. When the saliva flow rate is low, this shows risk to dental caries. Further, [21] explained how saliva flow measurement is an important risk assessment and management measure for dental caries.

### 3.1. Prevention of Caries

Dental caries can be prevented through various ways:

  Sealants: this can be done as [22] shows how sealants are introduced over the specific carious region to stop the decay process on the tooth. This prevents food particles from collecting in the tooth pits, thus preventing caries.
  Remineralization: this is done by hardening the tooth's enamel as explained by [23] to prevent dental caries from even taking place.
  Fluoride: this is found from excessive fluorosis in drinking water; in [24], a practicing dentist, associated with the enamel [3], showed its effects on the enamel of the tooth. Research from [25] gave an optimal level of fluoride in water to prevent dental caries.
  Risk assessment: this entails the prior determination of someone's developing dental caries during a specific period of time as [26] explained to enable easier management. Assessment is very useful in determining whether extra diagnostic measures are required. The work in [27] explained how assessment assesses effectiveness of previous caries control measures and acts as a guide to treatment planning in the future. Further research has seen that caries can be prevented by saliva when fluoride application is combined with regular removal of forming dental plaques, realized by brushing teeth. The research in [28] explained how saliva and small amounts of fluoride contributed to the hardening of the enamel and thus low risk of dental caries.

### 3.2. Diagnosis of Caries

Diagnosis of dental caries is done by the following:

  Clinical method: this is described by [29] and is explicitly defined as the visual detection of dental caries from an oral examination. They used separators to visualize areas of interest and also used dental floss applied on these areas to detect roughness of the surfaces.
  Radiographic method: the radiographs also known as X-rays are digital images and [30] introduced them in dentistry. The work in [31] further intensified the use of dental radiographs in their research to detect caries. The work in [32] generated images of higher diagnostic quality compared to those found from conventional films to detect caries in bitewing X-rays.

### 3.3. Treatment of Caries

Focus has shifted from surgical ways to the management of caries via restoration of tooth structure development or implants. The research work in [33] emphasized the prevention of the disease, remineralization steps, and minimizing the access to caries affected regions to avoid further decay.

## 4. Image Representation for Dental Segmentation and Detection

The representation of dental images is done through the segmentation of various regions of interest from the larger image to locate objects. Segmentation of images is, therefore, partitioning of an image into several segments to be used to identify objects and their edges. Image segmentation can be categorized according to similarity and discontinuity properties. Discontinuity based methods are referred to as boundary-based methods, while similarity-based methods are referred to as region-based methods [34].

Therefore, the segmentation process is based on dividing an image into groups of similar characteristics and features. Mathematically, segmentation of an image *R* is a finite set of regions *R*_1_ … *R*_*s*_. *R*=∪*i*=1*R*_*i*_*R*_*i*_∩*R*_*ji*_ ≠ *j*.

### 4.1. Categories of Dental Images Segmentation

Research done by [35, 36] further categorizes segmentation methods according to various characteristics such as region, entropy, shape, threshold, and pixels correlation among others. These characteristics were from thermal, X-ray images to aid analysis of specific points or regions of interest. Research studies show that dental image segmentation is classified as region-based, cluster-based, threshold-based, boundary-based, and watershed-based methods.

  Region-based: it divides an image into several regions based on discontinuities of pixel intensity. Reference [37] explained how segmentation of dental panoramic X-ray images assists dentists to detect osteoporosis disease. The work in [38] also used the region-based approach in segmentation of bitewing dental X-ray images.
  Threshold-based: this is done by choosing a threshold value from pixel intensities of an image. Then, pixels that exceed the threshold value are placed into a region, while those that are below the threshold value are placed in an adjacent region. Threshold-based segmentation is common in most of the reviewed articles [2, 6, 39–48].
  Cluster-based: it is the automatic grouping of image data based on certain degrees of similarity between the data. The degree of similarity depends on the problem being solved. The algorithm used to perform clustering of data uses the automatically detected groups as initial parameters. Research by [49] performed jaw lesion segmentation using the fuzzy C-means method. The work in [50] used a semifuzzy supervised clustering algorithm to segment dental radiographs.
  Boundary-based: it is used to find edge or point discontinuities on images. It detects color or pixel intensity discontinuities in the gray levels of the image. Active contours are used by [51–53], as one of the approaches to segment images based on their boundaries. The approach performs segmentation by outlining an object from an image and is also referred to as the snake method. Level set method (LSM) is another approach for detecting boundaries in an image. It handles segmentation by performing geometric operations to detect contours with topology changes. Examples of works on boundary-based segmentation are [54, 55], which used LSM to segment radiographic images.
  Watershed-based: it is performed on a grayscale image and used mathematical morphology to segment adjacent regions in an image; watershed-based segmentation was used by [56] on bitewing dental radiographs. It was also used by [57] as a combination of the K-means clustering and the watershed method for color-based segmentation.

### 4.2. Diagrammatic Representation of Dental Image Segmentation

**Figure 5:**
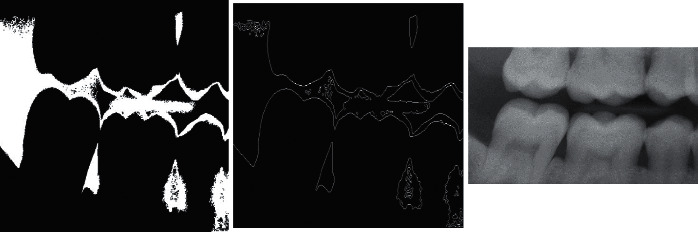
Left: region growing approach; central: canny edge detected image; right: tooth isolation and gap valley using binary intensity integral curves [38].

This section aims at giving pictorial representations of various segmentation methods, as can be viewed below in Figure 5. The work in [38] proposed a novel way of finding region of interests in both the gap valley and tooth isolation using edge intensity curves. It used the region growing approach [58] to detect the region of interest. It further used canny edge detection algorithm [59] to detect the edges of the isolated teeth.

**Figure 6:**
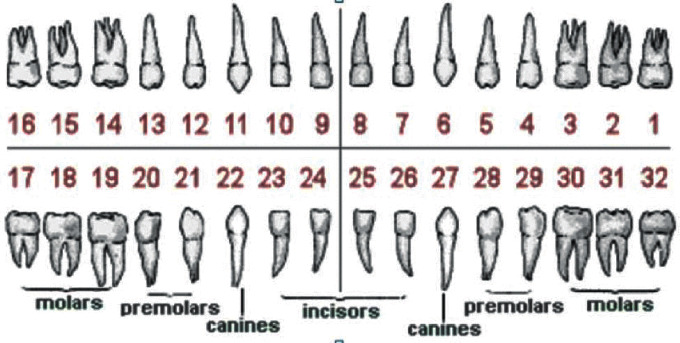
Dental universal numbering system [60].

A dental classification method by [60] proposed a classification of periapical images via the fuzzy value, which takes care of dental orientation problems such as missing teeth. They also used the dental universal numbering system to categorize teeth into incisors, canine, molar, and premolar as shown in Figure 6.

**Figure 7:**
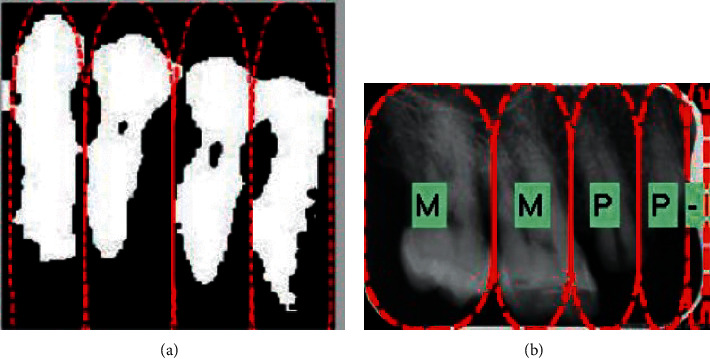
(a) Horizontal integral projection of lower tooth; (b) classification result [61].

Segmentation of the images is done through multiscale aggregation [61], which deals with pixel distortions in image data. Integral projection is used to detect horizontal individual teeth, and this is further classified according to one of the four incisor, molar, premolar, and canine categories.  Figure 7 shows images of the above-mentioned method.

**Figure 8:**
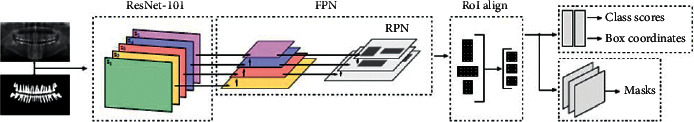
Training process of the segmentation system [63].

Additionally, [62] proposed the use of faster regions of a convolution neural network to detect and number periapical dental images. A filtering algorithm is used to delete overlapping boxes detected by the faster convolution network. A neural network is introduced to detect missing teeth. A rule-based teeth numbering system is used to match labels of detected teeth boxes to modify results that violate set rules. The work in [63] explained how they achieved instance segmentation through the use of a mask region-based convolution neural network. This system is an extension of [64], which includes a section of convolution networks to achieve the task of instance segmentation (Figure 8).

**Figure 9:**
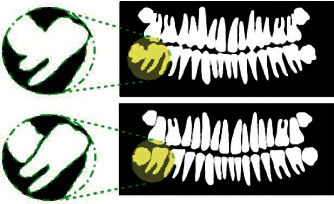
Process of separating the teeth.

Features are extracted from ResNet101, which then compose the feature pyramid network (FPN). The FPN defines anchors and extracts regions of interest (ROI). The region proposal network is formed from a combination of anchors and the feature pyramid network (FPN) (Figure 8). Finally, regions of interest are aligned to the same size and, further, each fixed size is classified as a tooth or a background (class scores). The fixed size features are localized by regression of bounding box coordinates. Finally, pixels are segmented by the full convolution network (FCN) [23] in each detected mask as seen in Figure 9.

Table 1 clearly shows related works grouped by various categories and methods. A diagnostic system proposed by [66] consists of Laplacian filtering, window-based adaptive threshold, morphological operations, statistical feature extraction, and back-propagation neural network. The back-propagation neural network is used to classify a tooth surface as normal or having dental caries. A study by [67] analyzes feature extraction performance of dental caries image using Gray Level Cooccurrence Matrix (GLCM) algorithm for contrasted two types of caries based on the theory of GV Black, namely, dental caries Class 3 and Class 4.

A CNN model using a U-shaped deep CNN (U-Net) proposed by [68] was for dental caries detection on bitewing radiographs, and it was further investigated whether the model can improve clinicians' performance.

## 5. Feature Extraction Techniques for Dental Images

Feature extraction on an image is done by the use of various methods depending on its texture, pixels, and color intensity. The method by [69] proposed evaluating the performance of various texture feature maps to recognize demineralization of caries. It used intraoral image analysis that includes run-length matrices (RLM), first-order features (FOF), cooccurrence matrices, gray tone matrices, local binary patterns, and K-means clustering to transform images of confirmed caries cases. The performance from the different feature maps was compared to that of radiographic images by several radiologists. Feature maps are a product of extraction of features from an image by various methods and techniques that include the following.

First-order features (FOF): this is a formalised description of the detection of probability of a particular intensity within data in an image, and this shape is used to determine image parameters such as contrast, sharpness, and other objects. For instance, for an image *I* with spatial resolution *W∗H* and range intensity *G*, its histogram is defined as

(1) $$\begin{array}{c} H\left(i\right)=\frac{1}{WH}\sum_{x=1}^{W}\sum_{y=1}^{H}\left\{\begin{array}{cc} 1, & I\left(x,y\right)=i\\ 0, & \text{otherwise} \end{array}\right\}i\in \left[0..G-1\right]. \end{array}$$

Several equations can be derived from the above equation, namely, mean, variance, and entropy, and can be represented as

(2) $$\begin{array}{c} FOF_{\text{mean}}=\frac{1}{WH}\sum_{x=1}^{W}\sum_{y=1}^{H}I\left(x,y\right), \end{array}$$

(3) $$\begin{array}{c} FOF_{\text{variance}}=\frac{1}{WH}{\sum_{x=1}^{W}\sum_{y=1}^{H}\left(I\left(x,y\right)-FOF_{\text{mean}}\right)}^{2}, \end{array}$$

(4) $$\begin{array}{c} FOF_{\text{entropy}}=\frac{1}{WH}-\sum_{i=1}^{G}H\left(i\right)\log\left(H\left(i\right)+\in \right). \end{array}$$

Run-length matrix is defined by changes in the illumination of pixel values on an image [70], to explain the coarse texture of pixels can be expressed by a larger section of similar color encoded as a matrix. Other works that have embraced this method include [71–73].

Gray level cooccurrence matrix (GLCM): this, according to [74], shows texture relations between adjacent pixels in the image texture. The matrix is calculated from entries that represent probabilities of coexisting gray tone pixels that are next to each other, and the distance between the pixels is a feature parameter. Other features that can be extracted by this matrix include contrast, entropy, variance, some average, and homogeneity. Related works include [75, 76].

Gray tone difference matrix (GTDM): this matrix, according to [77], is described as gray tone textural properties, which is the difference in pixel intensity level *I* and illumination *I* in a *K∗K* neighborhood described as

(5) $$\begin{array}{c} \text{GLTDM}\left(i\right)=\frac{1}{WH}\sum_{x=1}^{W}\sum_{y=1}^{H}\left|i-\bar{I}\right|\text{ where }\bar{I}\;=\frac{1}{M-1}, \end{array}$$

(6) $$\begin{array}{c} \sum_{m=-K}^{K}\sum_{n=-K}^{K}I\left(x+m,y+n\right),\left(m,n\right)\neq \left(0,0\right). \end{array}$$

Another method proposed by [78] combines GTDM, LBP, and K-means clustering for feature extraction.

Laws' texture energy measures: according to [79], a method that utilised set masks to calculate local energy of an image was proposed. These masks further detect textural characteristics such as edges, spots, levels, and ripples. Other works that use this method include [80, 81].

Local binary patterns (LBP): it detects small structures such as edges, lines, and spots [82], on the skin, thus representing them as binary patterns. For pixel inputs (*x*_*c*_, *y*_*c*_), a neighborhood of radius *R* and several evenly sampled points on radius *P* are specified. Local binary pattern function is given by

(7) $$\begin{array}{c} LBP_{P,R}\left(x_{c,y_{c}}\right)=\sum_{p=0}^{P-1}s\left[I\left(x_{c},y_{c}\right)-I\left(x_{p},y_{p}\right)\right].2^{p}. \end{array}$$

Here, s[.] operator returns 1 for positive values and 0 for negative values.

**Figure 10:**
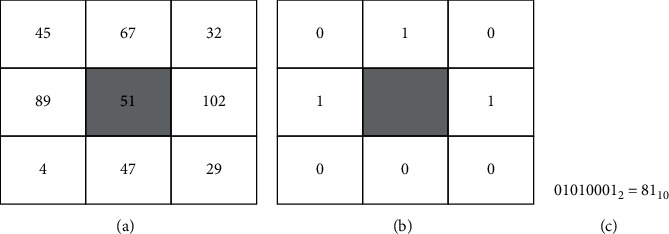
LBP operation given *P* = 1 and *R* = 1 [82].

Figure 10 shows a 3*∗*3 local binary operation concatenating all 8 bits to output a binary number, which is converted to a decimal number. This decimal number is the LBP code and is assigned to the center pixel. Other works that use LBP include [83] and [84].

**Figure 11:**
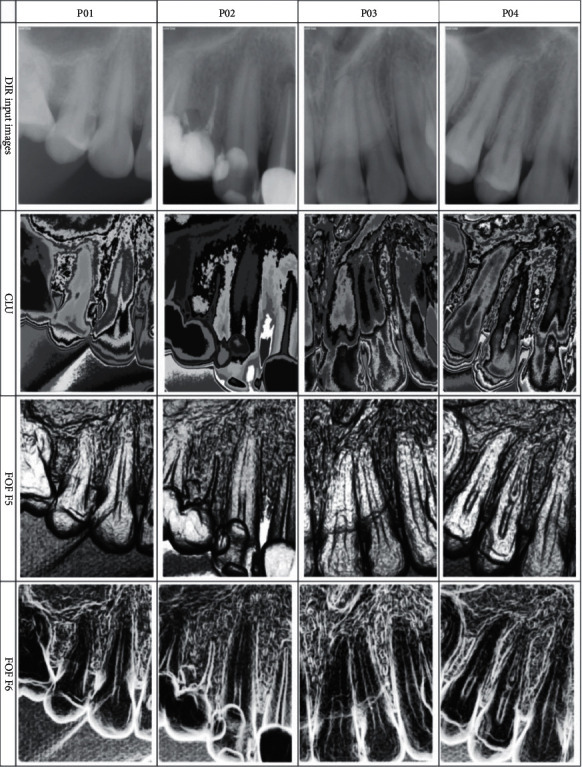
Intraoral radiographs texture feature maps [69].

Clustering: this used K-means method to cluster pixels. The intensity of pixels is taken into account; also prior information about the number of clusters should be defined. Euclidean distance is the metric used when adding individual pixels to the nearest cluster. A cluster value is set to the average value of all pixel intensities, and this procedure is iterated until a specified threshold is met [85]. Related works include [86–88].  Figure 11 shows the feature maps of the extraction techniques discussed.

According to [89], a textural feature system for diagnosis of dental caries in radiographs was used. This system introduced other feature extraction techniques such as the following.

Gabor filters: they were originally introduced by Gabor, D. (1946) and extracted edge-like components with very high frequency in a local region of an image. They are described as the best texture descriptor due to their use in segmentation, object recognition, tracking of motion, and image registration. Spatial domain Gabor is defined as

(8) $$\begin{array}{c} g\left(x,y\right)=\left(\frac{1}{2\pi \sigma_{x}\sigma_{y}}\right)\exp\left[\frac{1}{2}\left(\frac{x^{2}}{\sigma_{x}^{2}}+\frac{y^{2}}{\sigma_{y}^{2}}\right)+2\pi jWx\right]. \end{array}$$

Here *σ*_*x*_ and *σ*_*y*_ are standard deviations for (*x*, *y*) axis distribution, and the sinusoidal frequency is denoted by *W*. Other related studies on Gabor filters include [90–92].

Local ternary patterns: this, from [93], shows how LBP is extended to a three valued code from two values. The ternary code is got from comparing neighboring pixel values with a set threshold value *τ*. Values that are within the threshold value are set to 0, those above it are set to +1, and those below the threshold value are set to −1.

**Figure 12:**
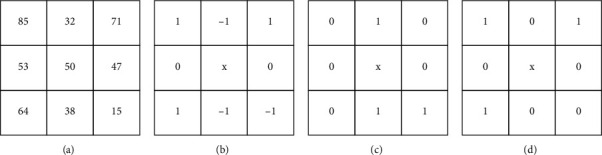
(a–d) LTP code with *τ* ≥ +15, *τ* ≤ −15 and both positive and negative LBP codes.

The threshold function value is defined as

(9) $$\begin{array}{c} f\left(x_{i},x_{c},\tau \right)=\left\{\begin{array}{cc} 1, & \text{if }x_{i}\geq x_{c}+\tau ,\\ 0, & \text{if}\left|x_{c}-x_{i}\right|<\tau ,\\ -1, & \text{if }x_{i}\leq x_{c}-\tau , \end{array}\right. \end{array}$$

where *τ* is the set threshold, *x*_*c*_ is the value of the central pixel, and *x*_*i*_ are the neighbouring pixels of *x*_*c*_. Diagrammatically, the process can be displayed by a Figure 12 LTP code for a 3 × 3 matrix image region. Other related works include [94–96].

Morphological gradient: the method introduced by [97] is used to increase the intensity of boundary edges of an image. This method makes it easier to observe edge boundaries and other objects clearly [98] and identify dental caries on teeth. Other works that involve the mMG method include [99–101]. Multiple morphological gradient consists of several encryption elements that aid in processing of images and include gradient, multiple values, and threshold.

Morphology gradient: it increases the intensity of the edges of objects in the image, and this is done by dilation subtracted by erosion morphology: (*A* ⊕ *B*) −  (*A*⊖*B*).

Multiplier value: it is a constant value to enable the increase in pixel value on the image. This is defined as

(10) $$\begin{array}{c} mv=\frac{\max\left(\max\left(A\right)\right)}{w}. \end{array}$$

Here *w* is the bit depth.

Threshold: this is done by separating pixel values into two classes, 0 and 1, which are dependent on a constant threshold value *q*. According to [99], thresholding is defined as

(11) $$\begin{array}{c} T\left(A\right)=\left\{\begin{array}{cc} 1, & A<q,\\ 0, & A\geq q. \end{array}\right. \end{array}$$

Here 0 < *q* ≤ *A*_max_.

Table 2 shows various works grouped by feature extraction method.

A novel deep convolution layer network (CNN) with a Long Short-Term Memory (LSTM) model was proposed by [103] for the detection and diagnosis of dental caries on periapical dental images.

## 6. Deep Learning Methods and Algorithms

Research by [104] explains how machine learning algorithms are divided into unsupervised and supervised learning algorithms.

### 6.1. Learning Algorithms

Supervised learning: the model is represented with a dataset *D*={*x*, *y*}_*n*=1_^*N*^ of input features *x* and *y*. They can take different forms depending on the learning task, for instance, with classification *y* will be a scalar representing a class label. With regression, it will take a vector of continuous variables. In dealing with a segmentation model, *y* can be a multidimensional label image; basically supervised training finds model parameters *θ* that best predict data given a loss function $L\left(y,\hat{y}\right)$ . This $\hat{y}$ describes the output of the model obtained by feeding *x* to *f*(*x*; ⊖) that represents the model.

Unsupervised learning: there are algorithms that are trained to find patterns, such as data without labels, for instance, clustering and principal component analysis methods. These algorithms can be performed on models with different loss functions.

### 6.2. Neural Networks

These are networks that contribute to deep learning systems. Neural networks consist of neurons with some activation *a* and parameters *θ*={*W*, *β*} , where *W* is weight and *β* is bias. Activation preselects the linear combination of input *x* to a neutron and its parameters, followed by element on element (.) for nonlinearity, referred to as the transfer function: *a*=*σ*(*w*^*T*^+*b*). Transfer functions for traditional neural networks are sigmoid and the hyperbolic tangent function. Multilayered neural networks known as perceptrons consist of several layers of these transformations:

(12) $$\begin{array}{c} f\left(x;\theta \right)=\sigma \left(W^{L}\theta \left(W^{L-1}\ldots \sigma \left(W^{0}x+b^{0}\right)+b^{L-1}\right)+b^{L}\right). \end{array}$$

Here *W*^*n*^ is a matrix comprising *w*_*k*_ rows with *k* as activation on the output. *n* is the number of current layers, and *L* is the final layer. Hidden layers are layers in between the input layer and the output layer. When a network contains multiple hidden layers, it is referred to as a deep neural network.

Activations done to the final layer which is the output layer of the network are mapped to a distribution over the number of classes *P*(*y|x*; *θ*) through a softmax function:

(13) $$\begin{array}{c} P\left(y|x;\theta \right)=\text{softmax }\left(x;\theta \right)=\frac{e^{{\left(W_{i}^{L}\right)}^{T}x+{\left(b_{i}\right)}^{L}}}{\sum_{k=1}^{K}e^{{\left(W_{k}^{L}\right)}^{T}x+{\left(b_{k}\right)}^{L}}}. \end{array}$$

Here *W*_*i*_^*L*^ is the weight of the output node associated with class *i*.

Stochastic gradient descent is the method used to fit parameters to dataset *D*. A small subset of the dataset is used to optimize maximum likelihood and this in return minimizes negative log-likelihood as

(14) $$\begin{array}{c} {\text{argmin}}_{\theta }-\sum_{n=1}^{N}\log\left[P\left(y_{n}|x_{n};\theta \right)\right]. \end{array}$$

This further leads to binary cross entropy loss for two class problems and categorical cross entropy for multiclass tasks. Deep neural networks gained popularity in 2006 and, in the method in [105], it was explained how DNNs were pretrained layer by layer and their stacked network fine-tuned to produce good evaluation performance. Currently, the most used deep learning popular networks are the convolution and the recurrent networks.

#### 6.2.1. Convolution Neural Networks

These networks have weights that are shared in a manner that the network performs convolutions on images. This means that there is no redundancy in the way the model learns separate detectors for the same object that occurs at different position on an image. It reduces the number of parameters to be used for learning. At each layer of the network, the image is convolved with a set of *K* kernels *W*=(*W*_1_ … *W*_*K*_) , with biases *β*=(*b*_1_ … *b*_*K*_) and each generating *X*_*k*_ feature map.

The features are subjected to a nonlinear transform *σ*(.) and the process iterated for every convolution layer *l* given by

(15) $$\begin{array}{c} X_{K}^{1}=\sigma \left(W_{K}^{l-1}*X^{l-1}+b_{k}^{l-1}\right). \end{array}$$

The convolution neural network also has pooling layers that aggregate pixels and their neighbors using an invariant function, which is the maximum operation. Aggregation is done to subsequent convolution layers and, at the end of the stream, a regular network layer is added where weights are not shared. The network is trained by feeding activations to the output layer through a softmax function. Under the CNNs, there exist common architectures that are widely used in the analysis of medical images and include the following.

General classification architectures: they include networks such AlexNet [106], which, unlike its precursor LeNet [107], consists of five convolution kernel layers employed in the input and output. AlexNet incorporated rectified linear units (ReLU) as their activation function, and this has become the most common choice in CNNs. Further, there has been an interest in using smaller kernels instead of single layers of kernels with a large receptive field; this in return has less number of parameters. Research by [108] discussed the use of deeper neural networks that have small fixed size kernels in each layer of the 19-layer model, referred to as VGG19 network model. More complex building blocks have been introduced to deep networks, to improve efficiency in the training process, and also reduce the amount of parameters used. There is also [109], where GoogLeNet is a 22-layered network that uses inception blocks with a set of convolutions of different sizes. The ResNet architecture was introduced and consists of ResNet blocks which, instead of learning a function, learn mappings in each layer that is close to the identity function [64]. There has been an increase in the use of these deep architectures due to their low memory use, and this even contributed to a recent version of GoogLeNet referred to as Inception v3 [110].

Multistream architectures: they are networks that accommodate multiple inputs in form of channels towards the input layer and then are later merged at any point in the network. Image processing can be done by the use of multiscale image analysis and classification for brain lesions [111]. Multistream architecture [112] is used for segmentation of natural images. Challenges of deep learning systems in the medical imaging domain are in adapting existing architectures, for instance, with different input formats (2D or 3D) data. Volumetric data can be divided into slices and fed as different streams to a network to avoid a result of large amounts of parameters. These techniques can still be used to perform knee cartilage segmentation [113]. The multistream architectures can be used for classification in the context of medical imaging [114].

Segmentation architectures: they are specific to the task of segmentation of medical images. CNNs are used to classify individual pixels with those in their neighborhood in an image. To avoid redundancy in the classification of the pixels, fully connected layers are rewritten as convolutions and this helps the CNN to take in input images larger than what it was trained on and produce a likelihood map. The resultant fully CNN (fCNN) can then be applied to an entire volume of images. This further leads to low resolution of output compared to the input images due to pooling of layers. There is a technique that applies the FCNN to shifted versions of the input, followed by stitching the result together to obtain a full resolution of the final output [115]. The FCNN was improved by proposing the U-net architecture that has convolutions in its downsampling and later an upsampling task to increase the image size [116]. Another method added skip connections to U-net architecture to connect downsampling and upsampling of the convolution layers [115]. A similar approach was used by [117], for 3D data, and [118] incorporated residual blocks and Dice loss layer to the U-net architecture instead of the commonly used cross entropy.

#### 6.2.2. Recurrent Neural Networks

These networks were developed for discrete sequence analysis and have varied lengths for both inputs and outputs, thus making them suitable for tasks such as machine translation. In a classification task, the model learns distribution over classes *P*(*y|x*_1_ … *x*_*T*_; *θ*) given sequence *x*_1_ … *x*_*T*_ as input.

The RNN has a hidden state *h* at time *t* which is the output of a nonlinear mapping from *x*_*t*_ and previous state *h*_*t*−1_: *h*_*t*_=*σ*(*Ex*_*t*_+*Rh*_*t*−1_+*b*). Here *W*, *R* are weight matrices that are shared over time. For a classification task, several fully connected layers are used and followed by a softmax to map the sequence over the classes as

(16) $$\begin{array}{c} P\left(y|x_{1}\ldots x_{T};\theta \right)=\text{softmax}\left(h_{T}:W_{\text{out}},b_{\text{out}}\right). \end{array}$$

RNNs have problems of memory shortage similar to those of other deep neural networks. Several techniques have been developed, such as the Long Short-Term Memory (LSTM) cell [119], to deal with problem. A simplification of the LSTM is the gated recurrent unit (GRU) [120]. RNNs are increasingly being adopted with promising results, for instance, [121], in the human brain challenge.

### 6.3. Unsupervised Models

Some unsupervised models reviewed include autoencoders, restricted Boltzmann machines, and deep belief networks.

#### 6.3.1. Autoencoders

These are networks trained to reconstruct input *x* on output $\overset{´}{y}$ via a hidden layer *h*. They have a weight matrix *W*_*x*,*h*_ and bias *b*_*x*,*h*_ from input to hidden state and *W*_*h*,*x*_ with corresponding bias *b*_*h*,*x*_ from hidden state to reconstruction. The hidden activation is computed as

(17) $$\begin{array}{c} h=\sigma \left(W_{x,h}x+b_{x,h}\right). \end{array}$$

Here, *h* is smaller than *x* to prevent the model from learning the identity function. According to [122], another solution was introduced to prevent the model from learning the identity function. The model uses a denoising autoencoder that trains the model to reconstruct input from noise. Deep autoencoders are realized by placing autoencoder layers on top of each other, and in most cases the layers were trained individually. Examples of autoencoders include the following.

Variational autoencoders: with these, [123] introduced the use of conditional variational autoencoders for pathology detection in medical images.

#### 6.3.2. Adverserial Networks

These are used for image generation tasks and include works from [124, 125].

#### 6.3.3. Deep Belief Networks

These are a type of Markov random field (MRF) which constitute an input layer *x*=(*x*_1_ … *x*_*N*_) and a hidden layer *h*=(*h*_1_ … *h*_*M*_) having a latent feature representation. Its connections are bidirectional and thus a generative model that can be sampled for new data points. An energy function can be defined for a state (*x*, *h*) of input and hidden layers as

(18) $$\begin{array}{c} E\left(x,h\right)=h^{T}W_{x}-c^{T}x-b^{T}h. \end{array}$$

Here *c* and *b* are the bias terms. Further, the probability of the system is given by

(19) $$\begin{array}{c} p\left(x,h\right)=\frac{1}{Z}\exp\left\{-E\left(x,h\right)\right\}, \end{array}$$

This makes computing the partition function *Z* intractable, while conditioned inference in computing *h* conditioned on *x* is tractable and is given by

(20) $$\begin{array}{c} P\left(h_{j}|x\right)=\frac{1}{1+\exp-b_{j}-W_{j}x}. \end{array}$$

The use of DBNs can be to fuse medical images [48]. DBNs also are used to extract high level features from medical images and effectively classify them [126].

### 6.4. Software and Hardware

**Figure 13:**
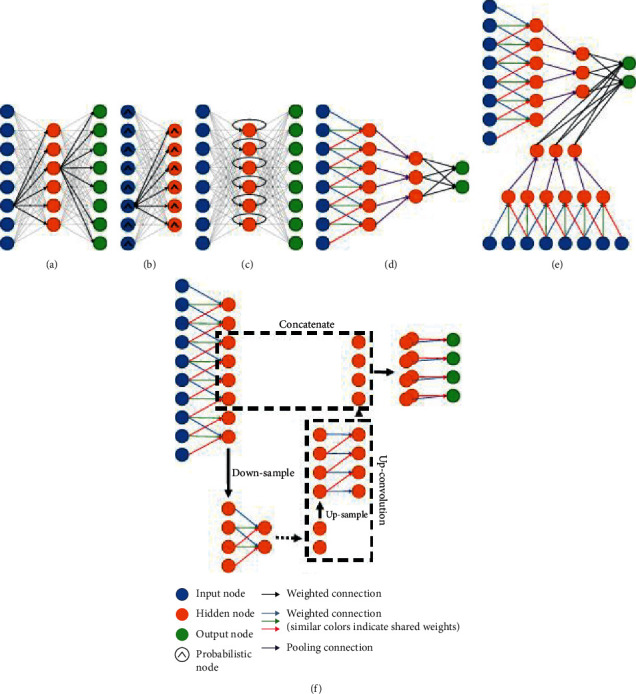
Representation of deep learning architectures. (a) Autoencoder. (b) Deep belief networks. (c) Recurrent neural network. (d) Convolutional neural network. (e) Multistream convolutional neural network. (f) U-net architecture [104].

These are the processors and software programs used to handle the running of various deep learning techniques. They include GPU computing libraries such as CUDA and OpenCL, these being very fast processor units compared to the previously used CPUs. These GPUs work hand in hand with the available open-source software programs that provide a platform to implement various operations of neural networks such as convolutions. Most popular packages include Caffe [127], TensorFlow [128], Theano [129], and Torch [130] that provide interfaces for implementing various operations in deep learning. They are also third-party packages written on top of frameworks like Keras [131] and GitHub [132].  Figure 13 shows a representation of deep learning architectures.

## 7. Deep Learning Uses in Medical Images

These systems can be used in various tasks like segmentation, classification, detection, and registration.

### 7.1. Classification

In this approach, one has a single input or multiple inputs with a single variable as output. For instance, in a disease classification setup, one has the disease or not and diagnosis of the disease is based on a sample of the dataset. Transfer learning is therefore realized and is defined as the use of pretrained networks to cover very large datasets for deep networks training. Transfer learning can be used to fine-tune a pretrained network on medical images and also for feature extraction on image data. These processes are beneficial in saving time used to train deep networks and enable extracted features to be analyzed faster. Object classification is usually done on a small part of the image and can be divided into two or more classes for analysis. For instance, [133] used three CNNs, each taking a nodule patch as input and each feature output is concatenated to form a final feature vector. Multistream CNNs are used to classify skin lesions, with each stream working on different resolutions of the image [134].

### 7.2. Detection

Detection of objects and their respective regions of interest in images is an important part of diagnosis by medical practitioners. The task consists of localization and identification of small regions in a full image. These systems are designed to automatically detect regions and decrease the reading time of human experts. An example of detection is [98], which explained the use of edge detection method to detect dental caries on dental images.

### 7.3. Segmentation

Segmentation of various organs allows deep analysis of several parameters related to volume and shape, for example, in skin or breast cancer analysis. It is always the first step before the detection process and is defined as the identification of the pixels which make up the interior or exterior contour of the object of interest. There have been a wide variety of methods developed to segment images using deep learning medical images. Segmentation is needed to perform accurate segmentation on 3D CNNs using multistream networks with different scales [135].

### 7.4. Registration

Registration is a common image analysis task in which coordinate transforms are calculated from one image to another. This is done iteratively assuming parametric transformations and a predetermined metric is optimized. Deep networks can benefit from registration by estimating a similarity measure for two images to drive an iterative optimization, for instance, [136]. They can also be used to predict transformation parameters using regression, for example, [137].

## 8. Databases Used by Dental Images

There is a need for efforts to build or come up with a public dataset to aid in developing of algorithms to be used in the dental imaging area. In order for this to come to pass, researchers need to release data used in their papers, and this will lead to a repository that can reliably catalogue and archive publicly dental imaging data. Several examples of datasets exist and include the digital database for screening mammography (DDSM) [138], which is used for mammogram image analysis and aids in screening of breast cancer. There exist several databases that are used in dental imaging and these include the following:

  PASCAL VOC 2007: this is from the results from the pascal visual object classes challenge of 2007 [139].
  Caltech101: this is a dataset of images to facilitate computer vision and its techniques [140]. This dataset is also applicable in image recognition and classification and contains a total of 9,146 images split into 101 distinct object categories.
  NORB: it is used for experiments in 3D object recognition shapes [141]. The dataset contains 50 toys belonging to five generic categories that include aeroplanes, cars, four legged animals, and trucks.
  CIFAR-10/100: this consists of 60000 32 × 32 color images in 10 classes, with 50000 training images and 10000 test images [142].
  MNIST: it is an acronym for modified national institute of standards and technology, and it is a dataset of handwritten digits commonly used for training and testing data in the machine learning field. It contains 60000 training images and 10000 testing images [143].
  LabelMe: it is a dataset and web based tool used for image annotation [144] and was created by MIT computer science intelligence laboratory (CSAIL). It is applicable in computer vision and is very dynamic, free, and open to public contribution. It contains 187240 images, 62197 annotated images, and 658992 labelled objects.
  ImageNet: it is a large dataset used for visual object recognition and has more than 20000 categories with each category containing several hundred images [145].
  Summary: both MNIST and CIFAR-10/100 datasets are available for the public, while the other datasets can be accessed by directly contacting the researchers. Dental datasets are difficult to find and thus researchers prefer using available public datasets. ImageNet is one of the datasets that is used for evaluations by several dental imaging models like ResNets and VGGNets.

## 9. Evaluation Protocols for Dental Images

Deep learning techniques are used on problems having very large datasets with thousands of instances, and therefore they need a way to estimate the performance of a given data configuration and use this for comparison with performances of other configurations. One of the ways is splitting data [146], since very large datasets require long training times.

### 9.1. Splitting Data

The data is split into training and testing data splits; for instance, Keras library for deep learning provides two ways of handling the splitting of data. It can split your data into a validation set and evaluate the performance of your model on that validation set. This is done by setting the validation split argument on the fit function to a certain percentage of your training dataset such as 30% for validation.

### 9.2. Manual K-Fold Cross Validation

This is used as the standard evaluation method for machine leaning techniques. This method splits the dataset into k-subsets and trains the model on all the subsets one after the other except one subset that is left out as the validation set. Evaluation is done on the left-out subset, which is the validation set, and the performance is averaged across all models created. The cross-validation method is applicable on small deep learning models and is used with 5 or 10 folds. We also have the method in [147] as the training-testing split method.

### 9.3. Training-Testing Split

Data is split into two parts, training set and testing set. A model is then fitted to the training set, and then the fit model is used to make predictions on the testing set. This is further used to evaluate the skill of the predictions and thus referred to as the training-testing split. Training-testing split is used as an estimate of how well the model performs on a dataset, especially when presented with new data. This method is preferred, especially with very large data and slow model to train. This is because the skill score for the model is noisy due to randomness of data. The randomness of data makes the model flexible but makes it less stable; for instance, you get different results from training the same model. This can be controlled by introducing a random seed and repeating experiments multiple times. The use of random seed is basically just using the same randomness every time the model is being fit and evaluated. Get an average of the estimated model skill after running the experiments multiple times. We also have [148] that also gives a description of the confusion matrix.

### 9.4. Confusion Matrix

It is an *N∗N* matrix with *N* number of classes being predicted and is used mostly with class output models. The matrix consists of several metrics that include the following:

  Accuracy: is the total number of correct predictions.
  Precision or positive predictive value: is a proportion of positive correct predictions.
  Negative predictive value: is a proportion of negative correct predictions.
  Recall or sensitivity: is a proportion of actual positive cases correctly identified.
  Specificity: is a proportion of actual negative cases correctly identified.

### 9.5. F1-Score

This is defined as the harmonic mean of the precision and recall values for a classification problem task.

The F1-score is given by

(21) $$\begin{array}{c} F_{1}={\left\{\frac{{\text{recall}}^{-1}+{\text{precision}}^{-1}}{2}\right\}}^{-1}=2.\frac{\text{precision}.\text{recall}}{\text{precision}+\text{recall}}. \end{array}$$

Harmonic mean is preferred over the arithmetic mean because it takes care of extreme values, for instance, those in a binary classification model. The F1-score can also be adjusted to increase effectiveness by adding *β* as an adjustable parameter to get

(22) $$\begin{array}{c} F_{\beta }=\left(1+\beta^{2}\right).\frac{\text{precision}.\text{recall}}{\left(\beta^{2}.\text{precision}\right)+\text{recall}}. \end{array}$$

## 10. Conclusion

From this survey, various techniques, methods, and approaches have been discussed concerning the segmentation and detection of dental images. Works that stem from the industry and academia have been mentioned and discussed, which include existing algorithms, segmentation and detection methods, databases, and various protocols for evaluating performance.

There is a huge potential for use of dental radiography and especially work focused on detection of dental images. Most of the existing systems dwell much on dental segmentation and not on feature extraction (detection). There is a need to improve existing dental detection systems, and one way to do so is by the introduction of automatic blob detection technique. Blob detection has been used in other fields of medical imaging but has not seen substantive use in the field of dental imaging. The use of such image analysis techniques to determine the presence of caries aims to create a system that takes a human diagnostic approach, whereby dental caries are diagnosed based on visual interpretation of teeth.

## 11. Future Work

There are issues and encouraging future perspectives of study which have popped out from our discussions here and they are highlighted as follows:

  Data availability and reliability: Deep learning networks require large amount of data to be able to achieve meaningful and effective performance results. Due to the nature of dental images, there is a need for hybrid datasets to aid good performance of the networks. There is a need for publicly available datasets for dental images to make deep learning in the field possible.
  Data standardization: Many methods discussed here are handling the preprocessing step through manual methods, such as cropping the region of interest on an image. These methods contribute to the loss of some key details from the images. Some networks end up dividing a whole image into subregions, and this slows down the learning process that occurs one subregion after the other. There are methods like downsampling which might lead to deletion of important details and this seems to have been due to limitations in computational power. Deep learning approaches have seen increased learning on whole images rather than the need for manual manipulation of images at the preprocessing stage in order to get more general and accurate results.
  Weight regularization methods: Deep learning networks can also be improved by introducing weight regularization to improve their performance. The regularization of weights involves optimization of model hyperparameters such as the learning rate and the dropout rate. Optimization of models at several key processes, such as the use of dropout at the segmentation stage, could improve the final result, and future work will look at how to implement such optimizations. Basically, weight regularization methods are introduced into networks for parameter optimization. Furthermore, introduction of early stopping regularization technique will also help in reducing overfitting, which is always a major problem with existing models.
  Hybrid approaches: Deep neural networks can also be achieved by combining several models or methods to form hybrid networks that will improve overall evaluation performance. The combination can be in any stage of the model, for instance, combining two preprocessing techniques to come with a single one to enhance image quality. This combination can also be handled by joining various attributes of different models to form one hybrid model that will enhance training, extraction, detection, and classification of objects. Combination of different convolution networks to form one hybrid network will be a good area to explore. This will save the long training and testing times that come with large networks having many convolution networks.

Oral healthcare follow-ups from medical practitioners are very important for risk assessment and management of dental caries [149–153].

## Figures and Tables

**Table 1:** Related works grouped by various categories and methods.

Category	Method	Related works
Region-based	Region growing approach	[37, 38]
Threshold-based	Histogram-based threshold	[2, 6, 39–47, 65]
Cluster-based	Fuzzy C-means	[49, 50]
Boundary-based	Active contours, level set method	[51–55]
Watershed-based	Watershed	[56, 57]

**Table 2:** Works grouped by feature extraction method.

Feature extraction method	Related works
FOF	[69]
Run-length matrix	[70–73]
LBP	[82–84]
Clustering	[85–88]
Laws of texture energy measures	(Laws, K., 1980) [80, 81]
GLCM	[74–76]
GTDM	[77, 78]
Gabor filters	[90–92, 102]
LTP	[93–96]
Multiple morphology gradient (mMG)	[98–101]

## Data Availability

Deep learning networks require large amount of data to be able to achieve meaningful and effective performance results. Due to the nature of dental images, there is a need for hybrid datasets to aid good performance of the networks. There is a need for publicly available datasets for dental images to make deep learning in the field possible
